# Improvement in aqueous solubility of achiral symmetric cyclofenil by modification to a chiral asymmetric analog

**DOI:** 10.1038/s41598-021-92028-y

**Published:** 2021-06-16

**Authors:** Junki Morimoto, Kazunori Miyamoto, Yuki Ichikawa, Masanobu Uchiyama, Makoto Makishima, Yuichi Hashimoto, Minoru Ishikawa

**Affiliations:** 1 Institute for Quantitative Biosciences, The University of Tokyo, 1-1-1 Yayoi, Bunkyo-ku, Tokyo, 113-0032 Japan; 2 Graduate School of Pharmaceutical Sciences, The University of Tokyo, 7-3-1 Hongo, Bunkyo-ku, Tokyo, 113-0033 Japan; 3 Advanced Elements Chemistry Laboratory, RIKEN Cluster for Pioneering Research (CPR), 2-1 Hirosawa, Wako-shi, Saitama, 351-0198 Japan; 4 Nihon University School of Medicine, 30-1 Oyaguchi-kamicho, Itabashi-ku, Tokyo, 173-8610 Japan; 5 Graduate School of Life Sciences, Tohoku University, 2-1-1, Katahira, Aoba-ku, Sendai, Miyagi 980-8577 Japan

**Keywords:** Chemical modification, Lead optimization

## Abstract

Decreasing the partition coefficient (Log*P*) by the introduction of a hydrophilic group is the conventional approach for improving the aqueous solubility of drug candidates, but is not always effective. Since melting point is related to aqueous solubility, we and other groups have developed alternative strategies to improve solubility by means of chemical modification to weaken intermolecular interaction in the solid state, thereby lowering the melting point and increasing the solubility. Here, we show that converting the symmetrical molecular structure of the clinically used estrogen receptor (ER) antagonist cyclofenil (**1**) into asymmetrical form by introducing an alkyl group enhances the aqueous solubility. Among the synthesized analogs, the chiral methylated analog (*R*)-**4c** shows the highest solubility, being 3.6-fold more soluble than **1** even though its hydrophobicity is increased by the methylation. Furthermore, (*R*)-**4c** also showed higher membrane permeability than **1**, while retaining a comparable metabolic rate, and equivalent biological activity of the active forms (*R*)-**13a** to **2**. Further validation of this strategy using lead compounds having symmetric structures is expected.

## Introduction

Aqueous solubility is an important physiochemical property of organic molecules, influencing ease of synthesis and purification, chemical and biological properties, functionality, effect on the environment, and so on^1,2^. From the viewpoint of drug discovery, the absorption of a drug by passive diffusion depends on the concentration gradient between the intestinal lumen and the blood, which is also influenced by solubility. Furthermore, efficacy evaluation and risk assessment of poorly soluble compounds are difficult. Thus, the aqueous solubility of drug candidates is regarded as a key physicochemical property^3^.

Thermodynamic solubility of a solid is defined as the concentration in solution when it is at equilibrium with the most stable crystal form^4,5^. In contrast, formulation strategies including crystal modification, particle size reduction can produce an increase in dissolution rate and a temporary increase of solubility^4,5^. However, it cannot produce a permanent alteration of solubility. Given sufficient time, the undissolved solute will revert to its most stable crystal form, and the solubility will approach the true thermodynamic solubility^6^. As a temporary increase of solubility by formulation strategies is not always achieved, poor solubility of drug candidates has been identified as the cause of numerous drug development failures. Thus, it would be better to generate drug candidates with sufficient thermodynamic solubility at the drug discovery stage.

The conventional and general method to increase aqueous solubility by chemical modification is introduction of hydrophilic group(s) into molecules to decrease the hydrophobicity (the common logarithm of partition coefficient, Log*P*_ow_). However, decreasing Log*P* is not necessarily effective to improve the properties of drug candidates. Oral drugs have to permeate the lipid bilayer membrane when they are absorbed from the intestinal lumen, so ideal drug candidates would have high hydrophobicity in addition to high aqueous solubility. Therefore, strategies to increase aqueous solubility without lowering hydrophobicity are required.

The solubility of a compound in water is also influenced by the molecular packing in the solid state^7,8^. Early studies to elucidate how the intermolecular interaction of organic compounds in the solid state affects their solubility in water were quite limited, although there is an old rule of thumb that organic compounds possessing weaker intermolecular interaction tend to show higher solubility in organic solvents. However, during the past decade, various strategies to improve the aqueous solubility of pharmaceutical compounds by means of chemical modification to disrupt intermolecular interactions have been developed^5,9–12^. Examples include disruption of intermolecular hydrogen bonds^13,14^, disruption of molecular planarity by ortho-substitution of biaryl groups^15–17^, bending molecular structure by changing the position of substituents^18–20^ (we had referred to this as disruption of molecular symmetry^9,12^, but “bending” is more appropriate because the examples shown in refs 9 and 12 possess the same point group after chemical modification), increasing the number of sp^3^-hybridized carbons^21,22^ (so-called “escape from flatland”), and introduction of a non-flat substituent at the *meta* position of a phenyl group^23^. We have shown that these strategies for disrupting intermolecular interactions can increase the aqueous solubility of molecules even if the hydrophobicity is concomitantly increased^9,12^. However, we sometimes encounter the cases that above molecular design strategies to increase the aqueous solubility are restricted to other parameters including permeability, biological activity, metabolism, promiscuous binding, safety, and so on. Therefore, the more choices of concrete molecular design strategies to increase the aqueous solubility, the possibility to the rational design of compounds satisfying all the parameters increases.

Among structurally simple compounds, compounds with higher symmetry tend to possess higher melting points^24–27^. Yalkowsky et al. reported that the higher the molecular symmetry number (σ), the higher the melting point tends to be, based on an analysis of 1200 structurally simple compounds^28^. In addition, in a cycloalkane having 3 to 10 carbon atoms, the boiling point increases with increase in the number of carbon atoms, but the melting point is relatively high for compounds having high symmetry at carbon numbers 3, 4 and 6^29^. Also, monosubstitution of unsubstituted benzene often leads to a decrease in melting point due to a decrease in symmetry. However, the relationship between symmetry and melting point does not generally hold for compounds with complex structures, such as many pharmaceutical compounds, and the relationship between structural symmetry and aqueous solubility remains to be established. Here, we report a new approach for improving aqueous solubility by chemical modification to change a symmetric molecular structure to an asymmetric molecular structure, illustrated by application to the estrogen receptor (ER) antagonist cyclofenil (**1**).

## Methods

### Physiochemical properties

#### Determination of melting point

Each compound was recrystallized twice from an appropriate solvent. The melting points of the crystals obtained in the two crystallizations were compared. If both crystals melted at the same temperature, this temperature was taken as the melting point. Recrystallization and melting point comparison was repeated until a consistent melting point was obtained.

#### Thermodynamic aqueous solubility of the most stable crystals

We assumed that crystals for which the melting point remained the same after repeated recrystallizations were the most stable crystal forms of the compounds, and we used them to evaluate aqueous solubility. The crystals were ground with an agate mortar and suspended in a mixture of 0.067 M phosphate buffer (pH 6.8) and EtOH (6:4). The suspension was shaken for 48 h at 4 °C, then filtered through a Millipore Millex-LG filter (0.20 μm). The filtrate was diluted in DMF and subjected to HPLC. The solubility was calculated by the absolute calibration curve method. We used a mixed solvent because the solubility in 0.067 M phosphate buffer (pH 6.8) was too low to allow quantification. EtOH is used clinically as a solubilizer for insoluble drugs such as taxol and pacritaxel. When the solutions were shaken at 37 °C, hydrolysis of the ester moiety occurred, and therefore the suspensions were shaken at 4 °C.

#### Membrane permeability

Membrane permeability was determined by HPLC on an IAM.PC.DD2 column (Regis Technologies, Inc., 10 μm, 300 Å, 30 mm × 4.6 mm) eluted with mobile phase consisting of 0.1 M phosphate buffer (pH 6.8) and CH_3_CN (4:6) at a flow rate of 1.0 mL/min, with UV monitoring at 254 nm, 37 °C.

## Results and discussion

**Figure 1:**
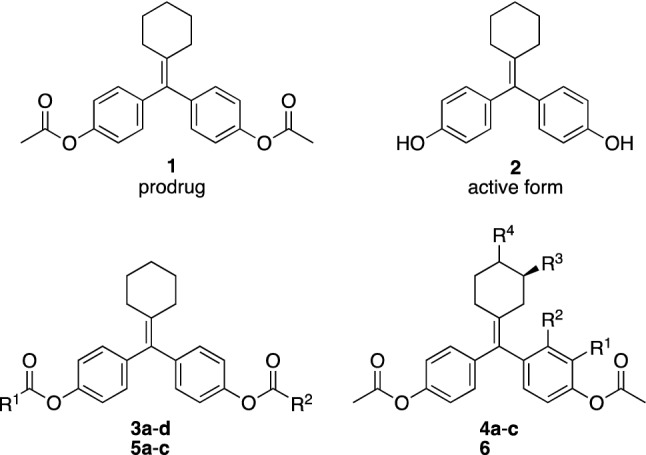
Chemical structures of cyclofenil (**1**), its active form **2**, asymmetric analogs **3a**–**d** and **4a**–**c**, and symmetric analogs **5a**–**c** and **6**.

Cyclofenil (**1**) is an antagonist of estrogen receptors (ER) α and β that is clinically used to treat menstrual disturbances, anovulatory infertility and menopausal symptoms (Fig. 1). However, **1** has poor aqueous solubility (< 1 μg/mL in 0.067 M phosphate buffer pH 7.4). We considered that this low solubility might be due to the symmetric structure (σ = 2, point group: C_2v_), and we hypothesized that chemical modification of **1** to an asymmetric structure (point group: C_s_ and C_1_) would improve the aqueous solubility. To investigate the relationship between molecular shape (symmetry) and aqueous solubility, we focused on a small chemical modification, that is, introduction of a hydrophobic alkyl group. Cyclofenil (**1**) is a prodrug that is hydrolyzed by esterases after administration, and the active form is diol **2**. Therefore, we initially considered that replacement of an acetyl group on **1** with a different acyl group (**3a**–**d**) might improve the aqueous solubility without loss of biological activity. As a second approach, we designed methylated analogs **4a**–**c** substituted at the 2-/3-position of the phenyl group or the 3-position of the cyclohexyl group, respectively. We also designed symmetrical analogs **5a**–**c** and **6** for comparison.

**Figure 2:**
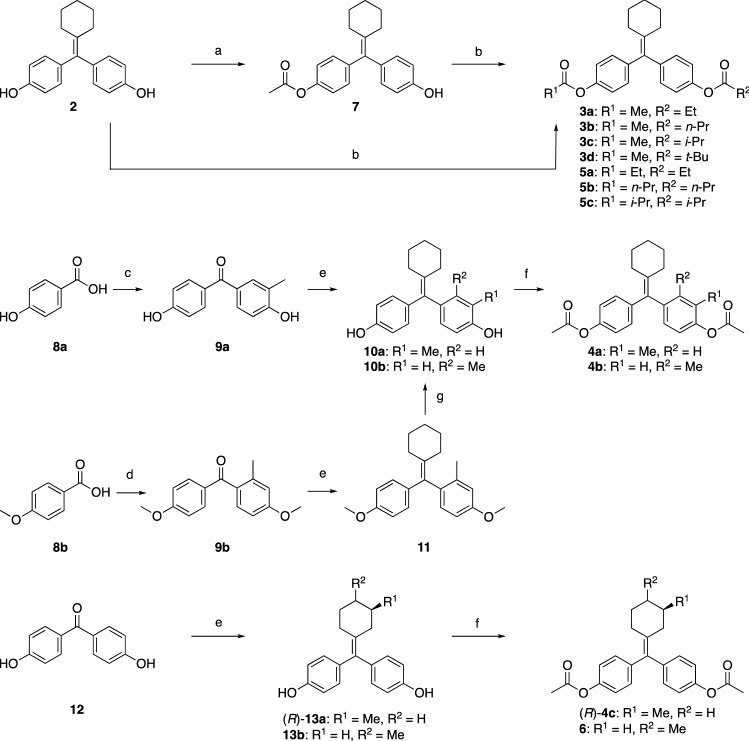
Reagents and conditions: (**a**) acetic anhydride (1.0 eq.), pyridine, DCM, 52% b.r.s.m.; (**b**) acid chlorides, pyridine, DCM or DCE, 80–87%; (**c**) *o*-cresol, ZnCl_2_, POCl_3_, 70 °C, 69%; (**d**) SOCl_2_, reflux, then *m*-cresol, AlCl_3_, DCM, 0 °C to rt, 70%; (**e**) cyclohexanones, Zn, TiCl_4_, THF, reflux, 75–91%; (**f**) acetic anhydride, pyridine, DCM, 75–95%; (**g**) BBr_3_, DCM, − 78 °C to rt, 66%.

Analogs of **1** were synthesized as shown in Fig. 2. Acetylation of one hydroxyl group of **2**^30^ with 1 equivalent of acetic anhydride afforded **7** along with **1**. Acylation of **7** and **2** with corresponding acyl chlorides gave asymmetric **3a**-**d** and symmetric **5a**–**c**, respectively. Friedel Crafts acylation between compounds **8a** and *o*-cresol, and **8b** and *m*-cresol gave **9a–b**, respectively. McMurry coupling reaction between **9a**/**9b**/**12** and cyclohexanones, including chiral (*R*)-3-hydroxyhexan-1-one, gave **10a**, **11**, (*R*)-**13a**, and **13b**, respectively. Diacetylation of **10a**-**b**, (*R*)-**13a** and **13b** gave **4a**-**b**, and (*R*)-**4c**, **6**, respectively.

We prepared the most stable crystals of **1**, **3a**–**d**, **4a–c**, **5a–c** and **6**, and measured their melting points, thermodynamic aqueous solubility (i.e., the solubility of the most stable crystal form to afford a saturated solution at equilibrium) and Log*P* (Table 1). Notably, asymmetric structural analogs **3b**, **4a**, **4b** and (*R*)-**4c** were more soluble than **1**. The melting points of these analogs were significantly lower than those of **1** and the other analogs. Log*P* values of all analogs were higher than that of **1**, as expected. These results indicate that the improvement in the aqueous solubility of **3b**, **4a**, **4b** and (*R*)-**4c** was not due to a decrease in hydrophobicity, but rather, was due to a decrease of intermolecular interaction in the crystals. The observation that the measured crystal densities of **3b**, **4a** and (*R*)-**4c** were lower than that of **1** supports this idea. On the other hand, **3a**, **3c** and **3d** showed decreased aqueous solubility compared to **1**, suggesting that an increase in hydrophobicity affects solubility more strongly than a decrease in intermolecular interaction.

**Table 1:** Physicochemical properties of cyclofenil analogs.

Compound	R^1^	R^2^	R^3^	R^4^	R^5^	R^6^	Aqueous solubility (μg/mL)^a^	Log*P*	Melting point (°C)	Crystal density (g/cm^3^)	Dihedral angle (°)^b^
**1**	Me	Me					7.76	4.91	137.5	1.268	55.3
**3a**	Me	Et					3.38	5.48	132.8		
**5a**	Et	Et					0.622	6.02	139.0		
**3b**	Me	*n*-Pr					10.8	6.00	69.0	1.236	
**5b**	*n*-Pr	*n*-Pr					0.964	7.08	80.9	1.244	
**3c**	Me	*i*-Pr					0.719	6.01	136.5		
**5c**	*i*-Pr	*i*-Pr					0.0811	7.16	129.0		
**3d**	Me	*t*-Bu					2.25	6.50	127.0		
**4a**			Me	H	H	H	10.3	5.26	114.0		54.4
**4b**			H	Me	H	H	23.6	5.12	99.9		66.1
(*R*)-**4c**			H	H	Me	H	27.8	5.34	92.0	1.227	
**6**			H	H	H	Me	3.61	5.41	137.0	1.243	

^a^Solubility in a mixture of 0.067 M phosphate buffer (pH 6.8) and EtOH (6:4).

^b^The most stable forms were estimated with Spartan’18.

The solubility of **4b** was higher than that of not only **1** but also **4a**, even though **4b** has almost the same hydrophobicity as **4a**. A plausible explanation for this is the difference of molecular planarity^9^. The melting point of **4b** was lower than that of **4a**, and the calculated dihedral angle between the cresol moiety and vinyl group in **4b** was larger than that of **4a** and **1**. These results indicate that introduction of a methyl group at the 2-position of the phenyl group of **1** disrupts the molecular planarity by increasing the dihedral angle, owing to steric hinderance between the methyl group and cyclohexyl group. Thus, not only introduction of asymmetry into the molecular structure, but also the disruption of planarity, appears to contribute to the increased aqueous solubility of **4b**.

The best result was obtained by introduction of a chiral methyl group as in (*R*)-**4c**, which showed the highest solubility among the compounds examined here, being 3.6 times soluble than **1**, together with the lowest crystal density. The reason for this is presumably the introduction of the chiral center, which results in greater molecular asymmetry. Considering conformational isomerization of the cyclohexyl group, the symmetry point groups of cyclofenil (**1**), **4a**/**4b** and (*R*)-**4c** are C_2v_, C_s_ and C_1_, respectively.

To exclude the possibility that the improvement in aqueous solubility was caused by increased molecular flexibility, which is known to be associated with a decrease of melting point^24^, we next compared the symmetrical and asymmetrical analogs. The solubility of each symmetrical compound **5a-c** was lower than that of the corresponding asymmetric compound **3a–c**. The symmetrical analog **6** also had lower solubility than the asymmetric isomer (*R*)-**4c.** Furthermore, comparison of the isomers **3b**/**5a** and **4a**/**4b**/(*R*)-**4c**/**6** clearly showed that asymmetric analogs **3b** (17.3-fold more soluble)/**4a** (2.9-fold)/**4b** (6.5-fold)/(*R*)-**4c** (7.7-fold) possess higher solubility and lower melting points than symmetric isomers **5a** and **6**. These results indicate that asymmetry contributes to the decrease in intermolecular interaction and the improvement of aqueous solubility.

Next, we investigated whether the synthesized compounds act as prodrugs, i.e., we measured their membrane permeability as prodrugs, the metabolic conversion of prodrugs into the active forms, and the biological activity of the active forms.

Firstly, we evaluated membrane permeability by using an Immobilized Artificial Membrane (IAM) column, which contains silica gel bearing phosphatidylcholine and widely utilized to predict membrane permeability^31,32^. As expected, the retention time of all asymmetric structural analogs was longer than that of **1** (Table 2), suggesting that the asymmetric structural analogs show higher membrane permeability due to increased hydrophobicity.

**Table 2:** retention time of cyclofenil analogs on IAM column.

Compound	Retention time (min)^a^
**1**	3.15
**3a**	4.22
**3b**	5.97
**3c**	5.93
**3d**	8.44
**4a**	3.72
(*R*)-**4c**	3.93

^a^IAM.PC.DD2 Column, mobile phase consisting of 0.1 M phosphate buffer (pH 6.8) in 60% CH3CN.

The metabolic rate of prodrugs was evaluated using human cryopreserved hepatocytes (Table 3). Although the metabolic rate of **3d** with a bulky acyl group was decreased, most of the asymmetric structural analogs, including those with improved aqueous solubility (**3b**, **4a**, (*R*)-**4c**), were metabolized to the active form as quickly as **1**. Thus, we concluded that the chemical modifications applied here had little effect on the metabolic rate.

**Table 3:** Metabolic rate of cyclofenil analogs.

Prodrug	Rate of active form (%)
**1**	100
**3a**	100
**3b**	92.1
**3c**	94.5
**3d**	77.7
**4a**	100
(*R*)-**4c**	100

Finally, we evaluated the ERα and ERβ antagonistic activities of the active forms **2**, **10a** and (*R*)-**13a** by means of luciferase reporter gene assay (Table 4). Under our assay conditions, **2**, which is the active form of not only **1** but also **3a-d**, showed antagonistic activities with IC_50_ values of 13 nM against ERα and 4.5 nM against ERβ. Compound **10a** showed decreased antagonistic activities, with IC_50_ values of 120 nM against ERα and 51 nM against ERβ. On the other hand, (*R*)-**13a** showed similar antagonistic activities to **2** against both ERα and ERβ. This results is consistent with the reported ERs antagonistic activity of racemic-**13a**^33^.

**Table 4:** ER-antagonistic activities of the active forms.

Active form	Antagonistic activity IC_50_ (nM)	
	ERα	ERβ
**2**	13 ± 7.5	4.5 ± 0.99
**10a**	120 ± 7.1	51 ± 40
(*R*)-**13a**	16 ± 0.71	4.5 ± 0.57

## Conclusion

The aqueous solubility of drug candidates is regarded as a key physicochemical property, and a greater number of concrete molecular design strategies to improve the aqueous solubility is required. This paper describes relationships between structural feature and solubility of a small molecular drug. And we developed a strategy for improving aqueous solubility by chemical modification to convert a symmetric molecular structure into an asymmetric molecular structure, focusing on cyclofenil (**1**). We found that a tiny molecular change, i.e. the introduction of an alkyl group to generate asymmetric analogs **3b**, **4a**, **4b** and (*R*)-**4c** increased the aqueous solubility in spite of the associated increase in hydrophobicity. Indeed, asymmetric analog **3b** was 17.3-fold more soluble than the corresponding symmetric isomer **5a**. The melting point and crystal density of **3b** and (*R*)-**4c** were lower than those of **1**, supporting the view that the improvement in aqueous solubility was due to the disruption of intermolecular interaction.

In this report, we carried out chemical modifications that intentionally increase hydrophobicity. This is because (1) to clarify the mechanisms of improvement of aqueous solubility, and (2) increase of both aqueous solubility and hydrophobicity is obviously tough. When the balance between hydrophobicity and aqueous solubility is important for compounds, we believe that this strategy would open up one possibility for molecular design. To enhance the utility of this strategy in the real medicinal chemistry, multiple strategies to improve aqueous solubility are likely to be superior to the single strategy. In fact, not only introduction of asymmetry into the molecular structure, but also the disruption of planarity appears to contribute to the further increased aqueous solubility of **4b**. In addition, introducing substituents would be selected depending on the Log *P* of lead compounds in the purpose of not only improving aqueous solubility but also optimizing hydrophobicity of drug candidates. Further validation of this strategy using lead compounds having symmetric structures is expected.

Simple methylation of **1** afforded the most soluble compound, (*R*)-**4c**, which was 3.6-fold more soluble than **1** even though its hydrophobicity is concomitantly increased. Furthermore, asymmetric (*R*)-**4c** is 7.7-fold more soluble than the corresponding symmetric isomer **6**, and (*R*)-**4c** showed better membrane permeability, a similar metabolic rate, and equivalent biological activity of the active forms (*R*)-**13a** to **2**. It is noteworthy that (*R*)-**4c** possesses a chiral center, which disrupts symmetry. Chiral pharmaceutical compounds are not necessarily desirable because of the difficulties of asymmetric synthesis and the need for additional quality control. However, it has been reported that the presence of chiral centers correlates with success in translation from discovery through clinical testing to drug approval^21^. In addition, it was recently reported that a benzene mimetic bearing a chiral center showed improved aqueous solubility, though its hydrophobicity was also decreased^35^. Our finding that a chiral compound showed better drug-like properties (aqueous solubility and membrane permeability) than achiral analogs highlights the importance of chiral compounds and asymmetric synthesis for medicinal chemistry^36,37^.

Medicinal chemists knows that methylation rarely leads to improved aqueous solubility^34^. And the mechanism of improved solubility by methylation is not fully understood. Our continuous works including this paper at least partially unveil the mechanism of improved aqueous solubility by methylation. And this work will provide one rational strategy for improvement of aqueous solubility by methylation into the specific position.

## Supporting Information

Methods including preparation of compounds, computational chemistry, and biology.

## Supplementary Information

Supplementary Information.

## Abbreviations

ER: Estrogen receptor
HPLC: High-performance liquid chromatography
