# Supplementary material for: Improvement in aqueous solubility of achiral symmetric cyclofenil by modification to a chiral asymmetric analog
Source: Sci Rep. 2021 Jun 16;11:12697. doi: 10.1038/s41598-021-92028-y (PMC8209153; doi:10.1038/s41598-021-92028-y)
Supplement: Supplementary file 1 — Supplementary Information. [file 41598_2021_92028_MOESM1_ESM.docx]

**Supporting Information**

**Improvement in aqueous solubility of achiral symmetric cyclofenil by modification to a chiral asymmetric analog**

Junki Morimoto^a^, Kazunori Miyamoto^b^, Yuki Ichikawa^a^, Masanobu Uchiyama^b,c^, Makoto Makishima^d^, Yuichi Hashimoto^a^, Minoru Ishikawa*^e^

^a^ Institute for Quantitative Biosciences, The University of Tokyo, 1-1-1 Yayoi, Bunkyo-ku, Tokyo 113-0032, Japan

^b^ Graduate School of Pharmaceutical Sciences, The University of Tokyo, 7-3-1 Hongo, Bunkyo-ku Tokyo 113-0033, Japan

^c^ Advanced Elements Chemistry Laboratory, RIKEN Cluster for Pioneering Research (CPR), 2-1 Hirosawa, Wako-shi, Saitama 351-0198, Japan

^d^ Nihon University School of Medicine, 30-1 Oyaguchi-kamicho, Itabashi-ku, Tokyo 173-8610, Japan

^e^ Graduate School of Life Sciences, Tohoku University, 2-1-1, Katahira, Aoba-ku, Sendai, Miyagi 980-8577, Japan

Corresponding author: Minoru ISHIKAWA

Graduate School of Life Sciences, Tohoku University, 2-1-1, Katahira, Aoba-ku, Sendai, Miyagi 980-8577, Japan

E-mail: minoru.ishikawa.e4@tohoku.ac.jp

**Chemistry**

**General conditions**

^1^H NMR and ^13^C NMR spectra were recorded on a JEOL ECX-500 spectrometer. Coupling constants are reported in hertz (Hz). The following abbreviations are used to designate the multiplicities: s = singlet, d = doublet, dd = double doublet, t = triplet, q = quartet, sext = sextet, sep = septet, m = multiplet. High-resolution mass spectra were acquired on a Bruker Daltonics micrOTOF-II in the positive or negative ion mode. Melting points were determined by using a Yanagimoto hot-stage melting point apparatus and are uncorrected. HPLC analyses were performed on Inertsil ODS-4 reversed-phase column (GL Science Inc., 5 μm, 4.6 mm x 150 mm) eluted with a mobile phase consisting of H_2_O and CH_3_CN.

**Bis(4-hydroxyphenyl)methylenecyclohexane (2).**

A two-necked, round-bottomed flask containing zinc powder (2400 mg, 36.0 mmol) was fitted with a reflux condenser, and charged with Ar gas. THF (20 mL) was added, the reaction mixture was cooled in an acetone bath (-20 °C), and then titanium (IV) chloride (3280 mg, 17.2 mmol) was added slowly. The reaction mixture was refluxed for 2 h at 70 °C and then cooled to room temperature. A solution of **12** (1000 mg, 4.66 mmol) and cyclohexanone (392 mg, 4.66 mmol) dissolved in THF (20 mL) was injected via a syringe. The mixture was refluxed for 2 h, then cooled, and slowly poured into a cooled NaHCO_3_ solution (150 mL). EtOAc (50 mL) was added with vigorous stirring. The heterogeneous solution was filtered through Celite. The organic layer was decanted, and the aqueous layer was extracted with additional EtOAc. The combined organic layer was dried over Na_2_SO_4_ and concentrated. Flash column chromatography (EtOAc/hexane = 3:7) of the residue gave **2** (989 mg, 75%) as a colorless solid. ^1^H NMR (500 MHz, DMSO-*d*_6_) δ 6.85 (d, *J* =8.5 Hz, 4H), 6.56 (d, *J* = 8.5 Hz, 4H), 2.14-2.16 (m, 4H), 1.50-1.55 (m, 6H). ^13^C NMR (125 MHz, DMSO-*d*_6_) δ 155.5, 136.3, 133.9, 133.7, 130.4, 114.6, 31.9, 28.2, 26.3. HRMS (ESI) calcd for C_19_H_20_O_2_ 279.1380; found 279.1381 (M-H)^−^.

**4-(Cyclohexylidene(4-hydroxyphenyl)methyl)phenylacetate (7).**

To a stirred solution of **2** (300 mg, 1.07 mmol) in DCM (3.0 mL), pyridine (101.2 mg, 1.28 mmol) and acetic anhydride (109.2 mg, 1.07 mmol) were added. The mixture was stirred for 16 h, and concentrated in vacuo. The residue was redissolved in EtOAc. The solution was washed with 2 M HCl aq, dried over Na_2_SO_4_ and concentrated in vacuo. Flash column chromatography (EtOAc/hexane) of the residue gave **1** (173 mg, 51% b.r.s.m) and **7** (155 mg, 52% b.r.s.m, colorless solid), together with recovered **2** (39 mg). ^1^H NMR (500 MHz, CDCl_3_) δ 7.10 (d, *J* =8.5 Hz, 2H), 6.93-6.98 (m, 4H), 6.70 (d, *J* = 9.0 Hz, 2H), 2.28 (s, 3H), 2.20-2.22 (m, 4H), 1.55-1.58 (m, 6H). HRMS (ESI) calcd for C_21_H_22_NaO_3_ 345.1461; found 345.1468 (M+Na)^+^.

**4-((4-Acetoxyphenyl)(cyclohexylidene)methyl)phenylpropionate (3a).**

To a solution of **7** (100 mg, 0.31 mmol) in DCM (2 mL), pyridine (219 mg, 1.86 mmol) and propionyl chloride (131 mg, 1.42 mmol) were added. The mixture was stirred for 23 h and concentrated in vacuo. The residue was redissolved in CHCl_3_. This solution was washed with 2 M HCl aq., dried over Na_2_SO_4_ and concentrated in vacuo. Flash column chromatography (EtOAc/hexane = 1:3) of the residue gave **3a** (106 mg, 87%) as a colorless solid, which was recrystallized from EtOAc/hexane = 2:25 (from 60 °C to r.t.). ^1^H NMR (500 MHz, CDCl_3_) δ 7.09-7.11 (m, 4H), 6.81-7.00 (m, 4H), 2.57 (q, *J* = 7.5 Hz, 2H), 2.28 (s, 3H), 2.23-2.23 (m, 4H), 1.57-1.59 (m, 6H), 1.25 (t, *J* = 7.5 Hz, 3H). ^13^C NMR (125 MHz, CDCl_3_) δ 173.1, 169.6, 149.2, 149.1, 140.5, 140.4, 140.4, 132.9, 131.0, 121.1, 32.6. 28.8, 27.9, 26.9, 21.3, 9.2. HRMS (ESI) calcd for C_24_H_26_NaO_4_ 401.1723; found 401.1728 (M+Na)^+^.

**4-((4-Acetoxyphenyl)(cyclohexylidene)methyl)phenylbutyrate (3b).**

To a solution of **7** (100 mg, 0.31 mmol) in DCE (2 mL), pyridine (95.2 mg, 0.93 mmol) and butyryl chloride (33.0 mg, 0.62 mmol) were added. The mixture was stirred for 10 h at 50 °C and concentrated in vacuo. The residue was redissolved in CHCl_3_ This solution was washed with 2 M HCl aq., dried over Na_2_SO_4_ and concentrated in vacuo. Flash column chromatography (EtOAc/hexane = 1:3) of the residue gave **3b** (101 mg, 80%) as a colorless solid, which was recrystallized from hexane (from 60 °C to 4 °C). ^1^H NMR (500 MHz, CDCl_3_) δ 7.09-7.11 (m, 4H), 6.97-6.99 (m, 4H), 2.52 (t, *J* = 7.5 Hz, 2H), 2.28 (s, 3H), 2.23-2.24 (m, 4H), 1.78 (sext, *J* = 7.5 Hz, 2H), 1.57-1.59 (m, 6H), 1.04 (t, *J* = 7.5 Hz, 3H). ^13^C NMR (125 MHz, CDCl_3_) δ 172.3, 169.7, 149.1, 149.1, 140.5, 140.4, 140.4, 132.9, 131.0, 130.9, 121.1, 121.0, 36.4, 32.6, 28.8, 26.9, 21.3, 18.6, 13.8. HRMS (ESI) calcd for C_25_H_28_NaO_4_ 415.1880; found 415.1883 (M+Na)^+^.

**4-((4-Acetoxyphenyl)(cyclohexylidene)methyl)phenylisobutyrate (3c).**

To a solution of **7** (100 mg, 0.31 mmol) in DCE (2 mL), pyridine (143 mg, 1.40 mmol) and isobutyryl chloride (99.1 mg, 0.93 mmol) were added. The mixture was stirred for 23 h and concentrated in vacuo. The residue was redissolved in CHCl_3_. The resulting solution was washed with 2 M HCl aq., dried over Na_2_SO_4_ and concentrated in vacuo. Flash column chromatography (EtOAc/hexane = 1:3) of the residue gave **3c** (102 mg, 84%) as a colorless solid, which was recrystallized from EtOAc/hexane = 8:15 (from 60 °C to r.t.). ^1^H NMR (500 MHz, CDCl_3_) δ 7.09-7.11 (m, 4H), 6.97-6.99 (m, 4H), 2.78 (sep, *J* = 7.0 Hz, 1H), 2.52 (t, *J* = 7.5Hz, 2H), 2.28 (s, 3H), 2.21-2.25 (m, 4H), 1.58-1.61 (m, 6H), 1.30 (d, *J* = 7.0 Hz, 6H). ^13^C NMR (125 MHz, CDCl_3_) δ 175.8, 169.6, 149.3, 149.1, 140.5, 140.4, 140.4, 132.9, 131.0, 130.9, 121. 1, 121.0, 34.3, 32.6, 32.6, 28.8, 26.9, 21.3, 19.1. HRMS (ESI) calcd for C_25_H_28_NaO_4_ 415.1880; found 415.1878 (M+Na)^+^.

**4-((4-Acetoxyphenyl)(cyclohexylidene)methyl)phenylpivalate (3d).**

To a solution of **7** (100 mg, 0.31 mmol) in DCE (2 mL), pyridine (158.2 mg, 1.55 mmol) and pivaloyl chloride (149.51 mg, 1.24 mmol) were added. The mixture was stirred for 10 h at 50 °C and concentrated in vacuo. The residue was redissolved in CHCl_3_. The solution was washed with 2 M HCl aq., dried over Na_2_SO_4_ and concentrated in vacuo. Flash column chromatography (EtOAc/hexane = 1:3) of the residue gave **3d** (111mg, 85%) as a colorless solid, which was recrystallized from EtOAc/hexane = 1:20 (from 60 °C to r.t.). ^1^H NMR (500 MHz, CDCl_3_) δ 7.09-7.10 (m, 4H), 6.96-6.99 (m, 4H), 2.29 (s, 3H), 2.21-2.25 (m, 4H), 1.56-1.62 (m, 6H), 1.34 (s, 9H). ^13^C NMR (125 MHz, CDCl_3_) δ 177.3, 169.7, 149.5, 149.0, 140.5, 140.4, 140.3, 132.9, 131.0, 130.9, 121.0, 121.0, 39.2, 32.6, 32.6, 28.8, 28.7, 27.3, 26.9, 21.3. HRMS (ESI) calcd for C_26_H_30_NaO_4_ 429.2036; found 415.2042 (M+Na)^+^.

**(Cyclohexylidenemethylene)bis(4,1-phenylene)dipropionate (5a).**

To a stirred solution of **2** (300 mg, 1.07 mmol) in DCE (4.0 mL), pyridine (328 mg, 3.21 mmol) and propanoyl chloride (248 mg, 2.68 mmol) were added. The mixture was stirred for 3 h at 50 °C and concentrated in vacuo. The residue was redissolved in CHCl_3_. This solution was washed with 2 M HCl aq., dried over Na_2_SO_4_ and concentrated in vacuo. Flash column chromatography (EtOAc/hexane = 1:5) of the residue gave **5a** (342 mg, 81%) as a colorless solid, which was recrystallized from EtOAc/hexane = 1:3 (from 60 °C to r.t.). ^1^H NMR (500 MHz, CDCl_3_) δ 7.10 (d, *J* = 8.5 Hz, 4H), 6.87 (d, *J* = 8.5 Hz, 4H), 2.58 (q, *J* = 7.0 Hz, 4H), 2.22-2.25 (m, 4H), 1.57-1.59 (m, 6H), 1.26 (t, *J* = 7.5 Hz, 6H). ^13^C NMR (125 MHz, CDCl_3_) δ 173.1, 149.1, 140.4, 140.3, 132.9, 130.9, 121.0, 32.6, 28.8, 27.9, 26.9. HRMS (ESI) calcd for C_25_H_28_NaO_4_ 415.1880; found 415.1888 (M+Na)^+^.

**(Cyclohexylidenemethylene)bis(4,1-phenylene)dibutyrate (5b).**

To a stirred solution of **2** (300 mg, 1.07 mmol) in DCE (4.0 mL), pyridine (328 mg, 3.21 mmol) and butyryl chloride (286 mg, 2.68 mmol) were added. The mixture was stirred for 3 h at 50 °C and concentrated in vacuo. The residue was redissolved in CHCl_3_. This solution was washed with 2 M HCl aq., dried over Na_2_SO_4_ and concentrated in vacuo. Flash column chromatography (EtOAc/hexane = 1:5) of the residue gave **5b** (367 mg, 82%) as a colorless solid, which was recrystallized from EtOAc/hexane = 1:30 (from 60 °C to r.t.). ^1^H NMR (500 MHz, CDCl_3_) δ 7.10 (d, *J* = 8.5 Hz, 4H), 6.81 (d, *J* = 8.5 Hz, 4H), 2.52 (t, *J* = 7.5 Hz, 4H), 2.22-2.24 (m, 4H), 1.78 (sext, *J* = 7.5 Hz, 4H), 1.56-1.59 (m, 6H), 1.04 (t, *J* = 7.5 Hz, 6H). ^13^C NMR (125 MHz, CDCl3) δ 172.3, 149.1, 140.4, 140.3, 132.9, 130.9, 121.1, 36.4, 32.6, 28.8, 26.9, 18.6, 13.8. HRMS (ESI) calcd for C_27_H_32_NaO_4_ 443.2193; found 443.2198 (M+Na)^+^.

**(Cyclohexylidenemethylene)bis(4,1-phenylene)bis(2-methylpropanoate) (5c).**

To a stirred solution of **2** (200 mg, 0.71 mmol) in DCE (4.0 mL), pyridine (255 mg, 2.50 mmol) and isobutyryl chloride (190 mg, 1.78 mmol) were added. The mixture was stirred for 1 h at 50 °C and concentrated in vacuo. The residue was redissolved in CHCl_3_. The solution was washed with 2 M HCl aq., dried over Na_2_SO_4_ and concentrated in vacuo. Flash column chromatography (EtOAc/hexane = 1:3) of the residue gave **5c** (257 mg, 86%) as a colorless solid, which was recrystallized from EtOAc/hexane = 1:5 (from 60 °C to r.t.). ^1^H NMR (500 MHz, CDCl_3_) δ 7.10 (d, *J* = 8.5 Hz, 4H), 6.79 (d, *J* = 8.5 Hz, 4H), 2.78 (sep, *J* = 7.0 Hz, 2H), 2.22-2.25 (m, 4H), 1.57-1.59 (m, 6H), 1.30 (d, *J* = 7.5 Hz, 12H). ^13^C NMR (125 MHz, CDCl_3_) δ 175.8, 149.3, 140.4, 140.3, 132.9, 130.9, 121.0, 34.3, 32.6, 28.8, 26.7, 19.1. HRMS (ESI) calcd for C_27_H_32_NaO_4_ 443.2193; found 415.2203 (M+Na)^+^.

**(4-Hydroxy-3-methylphenyl)(4-hydroxyphenyl)methanone (9a).**

To a mixture of *o*-cresol (1000 mg, 9.25 mmol) and **8a** (1405 mg, 10.2 mmol) were added anhydrous zinc chloride (5400 mg), and phosphorus oxychloride (5 mL). The reaction mixture was stirred for 4 h at 70 °C, then poured over ice and kept at 4 °C for 24 h. The precipitated solid was collected by filtration and washed with NaHCO_3_ aq. and H_2_O to afford **9a** (1607 mg, 69%) as a red solid. ^1^H NMR (500 MHz, DMSO-*d*_6_) δ 7.57 (d, *J* = 8.5 Hz, 2H), 7.48 (m, 1H), 7.40 (dd, *J* = 8.5 and 2.0 Hz, 1H), 6.85 (t, *J* = 8.5 Hz, 3H), 2.16 (s, 3H). HRMS (ESI) calcd for C_14_H_11_O_3_ 227.0703; found 227.0689 (M-H)^−^.

**4-(Cyclohexylidene(4-hydroxyphenyl)methyl)-2-methylphenol (10a).**

A two-necked, round-bottomed flask containing zinc powder (2288 mg, 35.0 mmol) was fitted with a reflux condenser, and charged with Ar gas. THF (20 mL) was added, the flask was cooled in an acetone bath (-20 °C), and then titanium (IV) chloride (2902 mg, 15.3 mmol) was added slowly. The reaction mixture was refluxed for 2 h at 70 °C and then cooled to room temperature. A solution of **9a** (1000 mg, 4.38 mmol) and cyclohexanone (429 mg, 4.38 mmol) dissolved in THF (20 mL) was injected via a syringe. The mixture was refluxed for 4.5 h, then cooled and slowly poured into cold NaHCO_3_ aq. (150 mL). EtOAc (50 mL) was added with vigorous stirring, and the heterogeneous solution was filtered through Celite. The organic layer was decanted and the aqueous layer was extracted with additional EtOAc. The combined organic layer was dried over Na_2_SO_4_ and concentrated. Flash column chromatography (EtOAc/hexane = 3:7) of the residue gave **10a** (1049 mg, 81%) as a colorless solid. ^1^H NMR (500 MHz, DMSO-*d*_6_) δ 6.83 (d, *J* = 8.5 Hz, 2H), 6.70 (s, 1H), 6.65-6.67 (m, 4H), 2.13-2.16 (m, 4H), 2.04 (s, 3H), 1.51-155 (m, 6H). 13C NMR (125 MHz, DMSO-*d*_6_) δ 155.5, 153.7, 136.1, 134.1, 133.8, 133.7, 131.5, 130.4, 127.7, 123.1, 114.6, 114.0, 32.0, 31.9, 28.2, 26.4, 16.1. HRMS (ESI) calcd for C_20_H_21_O_2_ 293.1536; found 293.1556 (M-H)^−^.

**4-((4-Acetoxy-3-methylphenyl)(cyclohexylidene)methyl)phenylacetate (4a).**

To a stirred solution of **10a** (200 mg, 0.68 mmol) in DCE (3.0 mL), pyridine (134 mg, 1.70 mmol) and acetic anhydride (139 mg, 1.36 mmol) were added. The mixture was stirred for 2 h at 50 °C and concentrated in vacuo. The residue was redissolved in EtOAc. This solution was washed with 2 M HCl aq., dried over Na_2_SO_4_ and concentrated in vacuo. Flash column chromatography (EtOAc/hexane = 1:4) of the residue gave **4a** (182 mg, 75%) as a colorless solid, which was recrystallized from EtOAc/hexane = 3:4 (from 60 °C to r.t.). ^1^H NMR (500 MHz, CDCl_3_) δ 7.10 (d, *J* = 8.5 Hz, 2H), 6.90-7.00 (m, 5H), 2.30 (s, 3H), 2.29 (s, 3H), 2.21-2.24 (m, 4H), 2.12 (s, 3H), 1.56-1.63 (m, 6H). ^13^C NMR (125 MHz, CDCl_3_) δ 169.7, 169.4, 149.0, 147.8, 140.7, 140.6, 140.1, 133.0, 132.5, 130.9, 129.5, 128.5, 121.3, 121.0, 32.6, 32.5, 28.8, 26.9, 21.3, 21.0, 16.4. HRMS (ESI) calcd for C_24_H_26_NaO_4_ 401.1723; found 401.1734 (M+Na)^+^.

**(4-Methoxy-2-methylphenyl)(4-methoxyphenyl)methanone (9b).**

Thionyl chloride (2343 mg, 19.7 mmol) was added to **8b** (1500 mg, 9.85 mmol). The reaction was refluxed for 4 h, then cooled to room temperature, and added to a stirred solution of 3-methoxytoluene (802 mg, 6.57 mmol) and AlCl_3_ (1752 mg, 13.14 mmol in DCM (5 mL) at 0 °C. The reaction mixture was stirred at room temperature for 1 h, then quenched with H_2_O and extracted with DCM. The combined organic layers were washed with 2 M NaOH aq., dried over Na_2_SO_4_ and concentrated in vacuo. Flash column chromatography (EtOAc/hexane = 1:10) of the residue gave **9b** (1184 mg, 70%) as a colorless oil. ^1^H NMR (500 MHz, CDCl_3_) δ 7.77 (d, *J* = 8.5 Hz, 2H), 7.30 (d, *J* =8.5 Hz, 1H), 6.93 (d, *J* = 8.5 Hz, 1H), 6.81(d, *J* = 2.0 Hz, 1H), 3.88 (s, 3H), 3.85 (s, 3H), 2.36 (s, 3H). ^13^C NMR (125 MHz, CDCl_3_) δ 196.8, 163.5, 161.1, 139.9, 132.6, 131.5, 131.3, 116.7, 11379, 110.3, 55.6, 55.4, 20.7.

**1-(Cyclohexylidene(4-methoxyphenyl)methyl)-4-methoxy-2-methylbenzene (11).**

A two-necked, round-bottomed flask containing zinc powder (2039 mg, 31.2 mmol) was fitted with a reflux condenser, and charged with Ar gas. THF (20 mL) was added, the flask was cooled in an acetone bath (-20 °C), and then titanium (IV) chloride (2598 mg, 13.7 mmol) was added slowly. The reaction mixture was refluxed for 2 h at 70 °C and then cooled to room temperature. A solution of **9b** (1000 mg, 3.90 mmol) and cyclohexanone (383 mg, 3.90 mmol) dissolved in THF (10 mL) was injected via a syringe. The reaction mixture was refluxed for 2 h, then cooled, and slowly poured into cold NaHCO_3_ aq. (100 mL). EtOAc (50 mL) was added with vigorous stirring, and the heterogeneous solution was filtered through Celite. The organic layer was decanted, and the aqueous layer was extracted with additional EtOAc. The combined organic layer was dried over Na_2_SO_4_ and concentrated. Flash column chromatography (EtOAc/hexane = 1:30) of the residue gave **11** (1124 mg, 89%) as a colorless solid. ^1^H NMR (500 MHz, CDCl_3_) δ 7.05 (d, *J* = 8.5 Hz, 2H), 7.01 (d, *J* = 9.0 Hz, 1H), 6.79 (d, *J* = 8.5 Hz, 2H), 6.70-6.69 (m, 2H), 3.78 (s, 3H), 3.77 (s, 3H), 2.04-1.95 (m, 2H), 1.67-1.56 (m, 4H), 1.54-1.49 (m, 2H). ^13^C NMR (125 MHz, CDCl_3_) δ 158.2, 157.7, 138.9, 137.7, 135.7, 134.7, 132.4, 131.0, 130.8, 115.4, 113.2, 110.8, 55.3, 55.2, 32.8, 31.7, 28.8, 28.5, 27.0, 20.3.

**4-(Cyclohexylidene(4-hydroxyphenyl)methyl)-3-methylphenol (10b)**

Under an Ar atmosphere, a solution of **11** (1000 mg, 3.10 mmol) in dry DCM (7 mL) was cooled to -78 °C, and BBr_3_ (9.30 mL of 1 M in DCM, 9.30 mmol) was added. The mixture was stirred for 2 h at room temperature and then quenched with water in an ice bath and extracted with EtOAc. The combined organic layer was washed with water, dried over Na_2_SO_4_ and concentrated. Flash column chromatography (EtOAc/hexane = 1:3) of the residue gave **10b** (602 mg, 66%) as a colorless solid. ^1^H NMR (500 MHz, DMSO-*d*_6_) δ 6.86 (d, *J* = 8.5 Hz, 2H), 6.81 (d, *J* = 9.0 Hz, 1H), 6.65 (d, *J* = 9.0 Hz, 2H), 6.54-6.52 (m, 2H), 2.27-2.19 (m, 2H), 1.98 (s, 3H), 1.90 (t, *J* = 6.0 Hz, 2H), 1.54 (m, 4H), 1.44 (m, 2H). ^13^C NMR (125 MHz, DMSO- *d*_6_) δ 155.6, 155.4, 136.9, 136.4, 133.5, 132.6, 132.4, 130.3, 130.1, 116.4, 114.5, 112.4, 32.1, 31.0, 28.2, 27.9, 26.3, 19.7.

**4-((4-Acetoxy-2-methylphenyl)(cyclohexylidene)methyl)phenylacetate (4b)**

To a stirred solution of **10b** (400 mg, 1.36 mmol) in DCM (5.0 mL), pyridine (932 mg, 8.16 mmol) and acetic anhydride (646 mg, 8.16 mmol) were added. The mixture was stirred for 4 h at room temperature and concentrated in vacuo. The residue was redissolved in DCM. This solution was washed with 2 M HCl aq., dried over Na_2_SO_4_ and concentrated in vacuo. Flash column chromatography (EtOAc/hexane = 1:6) of the residue gave **4b** (488 mg, 95%) as a colorless solid, which was recrystallized from EtOAc/hexane = 1:20 (from 60 °C to r.t.). ^1^H NMR (500 MHz, CDCl_3_) δ 7.12 (d, *J* = 8.5 Hz, 2H), 7.10 (d, *J* = 9.0 Hz, 1H), 6.96 (d, *J* = 8.5 Hz, 2H), 6.89-6.87 (m, 2H), 2.34-2.31 (m, 2H), 2.27 (s, 6H), 2.12 (s, 3H), 2.04-1.94 (m, 2H), 1.66-1.56 (m, 4H), 1.55-1.47 (m, 2H). ^13^C NMR (125 MHz, CDCl_3_) δ 169.7, 169.6, 149.3, 148.9, 140.5, 140.1, 139.1, 138.0, 131.6, 131.0, 130.7, 122.9, 120.9, 118.6, 32.8, 31.6, 28.7, 28.5, 26.9, 21.3, 21.3, 20.2.

**(*R*)-4,4’-((3-Methylcyclohexylidene)methylene)diphenol ((*R*)-13a).**

A two-necked, round-bottomed flask containing zinc powder (1200 mg, 18.0 mmol) was fitted with a reflux condenser, and charged with Ar gas. THF (10 mL) was added, the mixture was cooled in an acetone bath (-20 °C), and then titanium (IV) chloride (1640 mg, 8.6 mmol) was added slowly. The reaction mixture was refluxed for 2 h at 70 °C and then cooled to room temperature. A solution of **12** (500 mg, 2.33 mmol) and (*R*)-(+)-3-methylcyclohexanone (261 mg, 2.33 mmol) dissolved in THF (20 mL) was injected via a syringe. The reaction mixture was refluxed for 4.5 h, then cooled, and slowly poured into cold NaHCO_3_ aq. (100 mL). EtOAc (50 mL) was added with vigorous stirring, and the heterogeneous solution was filtered through Celite. The organic layer was decanted and the aqueous layer was extracted with additional EtOAc. The combined organic layer was dried over Na_2_SO_4_ and concentrated. Flash column chromatography (EtOAc/hexane = 1:2) of the residue gave (*R*)-**13a** (625 mg, 91%) as a colorless solid. ^1^H NMR (500 MHz, DMSO-*d*_6_) δ 6.81-6.83 (m, 4H), 6.65-6.67 (m, 4H), 2.42 (t, *J* = 9.5 Hz, 2H), 1.80 (td, *J* = 13.0 and 2.0 Hz, 1H), 1.72 (d, *J* = 10.5 Hz, 2H), 1.45-1.58 (m, 2H), 1.26-1.34 (m, 1H), 1.03-1.12 (m, 1H), 0.83 (d, *J* = 6.5 Hz, 3H). ^13^C NMR (125 MHz, DMSO-*d*_6_) δ 155.5, 135.7, 134.1, 133.8, 133.7, 130.4, 130.4, 114.6, 34.7, 34.0, 31.4, 27.0, 22.3. HRMS (ESI) calcd for C_20_H_21_O_2_ 293.1536; found 293.1560 (M-H)^−^.

**(*R*)-((3-Methylcyclohexylidene)methylene)bis(4,1-phenylene)diacetate ((*R*)-4c).**

To a stirred solution of compound (*R*)-**13a** (200 mg, 0.68 mmol) in DCE (3.0 mL), pyridine (188 mg, 2.38 mmol) and acetic anhydride (208 mg, 2.04 mmol) were added. The mixture was stirred for 3 h at 50 °C and concentrated in vacuo. The residue was redissolved in CHCl_3_. The solution washed with 2 M HCl aq., dried over Na_2_SO_4_ and concentrated in vacuo. Flash column chromatography (EtOAc/hexane = 1:4) of the residue gave (*R*)-**4c** (232 mg, 80%) as a colorless solid, which was recrystallized from EtOAc/hexane = 1:60 (from 60 °C to r.t.). ^1^H NMR (500 MHz, CDCl_3_) δ 7.08-7.10 (m, 4H), 6.98-7.00 (m, 4H), 2.52 (d, *J* = 13.0 Hz, 2H), 2.29 (s, 3H), 2.28 (s, 3H), 1.85(td, *J* = 13.0 and 3.5 Hz, 1H), 1.77- 1.81 (m, 2H), 1.50-1.64 (m, 2H), 1.33-1.41 (m, 1H), 1.09-1.16 (m, 1H), 0.88 (d, *J* =6.5 Hz, 3H). ^13^C NMR (125 MHz, CDCl3) δ 169.7, 149.1, 140.7, 140.5, 139.8, 133.1, 131.0, 131.0, 121.1, 40.7, 35.3, 34.8, 32.0, 27.6, 22.5, 21.3. HRMS (ESI) calcd for C_24_H_26_NaO_4_ 401.1723; found 401.1728 (M+Na)^+^.

**4,4’-((4-Methylcyclohexylidene)methylene)diphenol (13b).**

A two-necked, round-bottomed flask containing zinc powder (1200 mg, 18.0 mmol) was fitted with a reflux condenser, and charged with Ar gas. THF (10 mL) was added, the mixture was cooled in an acetone bath (-20 °C), and then titanium (IV) chloride (1640 mg, 8.6 mmol) was added slowly. The reaction mixture was refluxed for 2 h at 70 °C and then cooled to room temperature. A solution of **12** (500 mg, 2.33 mmol) and 4-methylcyclohexanone (261 mg, 2.33 mmol) dissolved in THF (20 mL) was injected via a syringe. The reaction mixture was refluxed for 2 h, then cooled, and slowly poured into cold NaHCO_3_ aq. (100 mL). EtOAc (50 mL) was added with vigorous stirring, and the heterogeneous solution was filtered through Celite. The organic layer was decanted and the aqueous layer was extracted with additional EtOAc. The combined organic layer was dried over Na_2_SO_4_ and concentrated. Flash column chromatography (EtOAc/hexane = 1:2) of the residue gave **13b** (621 mg, 91%) as a colorless solid. ^1^H NMR (500 MHz, DMSO-*d*_6_) δ 6.82 (d, *J* = 8.5 Hz, 2H), 6.66 (d, *J* = 8.5 Hz, 2H), 2.46 (d, *J* = 13.5 Hz, 2H), 1.88 (td, *J* = 13.5 and 3.5 Hz, 2H), 1.69-1.72 (m, 2H), 1.53-1.60 (m, 1H), 0.95-1.03 (m, 2H), 0.89 (d, *J* = 6.0 Hz, 3H). ^13^C NMR (125 MHz, DMSO-*d*_6_) δ 155.6, 135.9, 134.0, 133.8, 130.4, 114.6, 36.4, 32.2, 31.2, 22.0. HRMS (ESI) calcd for C_20_H_21_O_2_ 293.1536; found 293.1539 (M-H)^−^.

**((4-Methylcyclohexylidene)methylene)bis(4,1-phenylene)diacetate (6).**

To a stirred solution of **13b** (300 mg, 1.02 mmol) in DCE (5.0 mL), pyridine (282 mg, 3.57 mmol) and acetic anhydride (312 mg, 3.06 mmol) were added. The mixture was stirred for 3 h at 50 °C and concentrated in vacuo. The residue was redissolved in CHCl_3_. This solution was washed with 2 M HCl aq., dried over Na_2_SO_4_ and concentrated in vacuo. Flash column chromatography (EtOAc/hexane = 1:3) of the residue gave **6** (343 mg, 88%) colorless solid, which was recrystallized from EtOAc/hexane = 3:8 (from 60 °C to r.t.). ^1^H NMR (500 MHz, CDCl_3_) δ 7.10 (d, *J* =8.5 Hz, 2H), 6.69 (d, *J* = 8.5 Hz, 2H), 2.56 (d, *J* = 7.0 Hz, 2H), 2.28 (s, 6H), 1.94 (td, *J* = 13.0 and 4.0 Hz, 2H), 1.74-1.78 (m, 2H), 1.58-1.65 (m, 1H), 1.01-1.10 (m, 1H), 0.92 (d, *J* = 6.5 Hz, 3H). ^13^C NMR (125 MHz, CDCl_3_) δ 169.7, 149.1, 140.6, 140.0, 133.0, 131.0, 121.1, 36.9, 32.8, 31.9, 22.1, 21.3. HRMS (ESI) calcd for C_24_H_26_NaO_4_ 401.1723; found 401.1736 (M+Na)^+^.

**X-ray crystallography**

**Crystal data**

Crystal data for **1**: C_23_H_24_O_4_, colorless plate crystal, triclinic, *a*=11.22997(2) Å, *b*=13.0412(3) Å, *c*=13.4031(3) Å, *α*=101.265(2)°, *β*=91.958(2)°, *γ*=96.654(2)°, V=1908.83 Å^3^. CCDC 2058201

Crystal data for **3b**: C_25_H_28_O_4_, colorless crystal, monoclinic, *a*=19.6645(8) Å, *b*=10.0846(3) Å, *c*=22.6923(10) Å, *α*=90°, *β*=110.771(5)°, *γ*=90°, V=4207.6 Å^3^. CCDC 2058202

Crystal data for (*R*)-**4c**: C_24_H_26_O_4_, colorless crystal, triclinic, a=10.0047(4) Å, b=10.8840(4) Å, c=11.3755(5) Å, α=107.121◦, β=102.692(4)◦, γ=111.449(4)◦, V=1024.48 Å^3^. CCDC 2058204

Crystal data for **5b**: C_27_H_32_O_4_, colorless columnar crystal, monoclinic, *a*=24.7138(6) Å, *b*=10.24810(10) Å, *c*=20.3569(5) Å, *α*=90°, *β*=119.421(3)°, *γ*=90°, V=4490.86 Å^3^. CCDC 2058203

Crystal data for **6**: C_24_H_26_NaO_4_, colorless plate crystal, monoclinic, *a*=12.8202(3) Å, *b*=8.1477(2) Å, *c*=38.7256(10) Å, *α*=90°, *β*=90.599(2)°, *γ*=90°, V=4044.87 Å^3^. CCDC 2058205

**Computational chemistry**

**Measuring of dihedral angle**

The most stable molecular conformations of **1**, **4a** and **4b** were calculated with Spartan’18 (Wavefunction, Inc.). The dihedral angles of the most stable molecule conformations obtained by molecular mechanics calculation (MMFF) and density functional theory (DFT) calculation (ωB97X-D/6-31+G*) were determined. As the measure of planarity, we used the average value of the four dihedral angles shown in Figure S1. When the absolute value of the dihedral angle was larger than 90 degrees, the value was corrected by subtracting 90 degrees.

**Figure S1.** Four dihedral angles used for calculating molecular planarity.

**Biology**

**Cell culture**

HEK 293 cells were cultured in DMEM supplemented with 10% FBS and penicillin and streptomycin at 37 °C in a humidified incubator (5% CO_2_).

**Reporter gene assay**

HEK 293 cells were plated at 20% confluence in a 96-well plate at 24 h prior to transfection. Transfection of plasmids (CMX-GAL4N-human ERα/β ligand binding domain, TK-MH100x4-Luc, CMX-β-galactosidase) was performed by the calcium phosphate coprecipitation method. Test compounds in DMSO (final 0.5%) were added at 24 h after transfection, together with 0.3 nM estradiol. The plate was incubated for 24 h, then luciferase substrate solution was added to each well and luminescence was measured on an EnVision microplate reader. β-Galactosidase was added and the absorbance was measured on the microplate reader with emission detection at 405 nm. Each sample was evaluated in triplicate for two times and the mean value was calculated. A six-point sigmoidal dose-response curve was generated for each compound. The IC_50_ value for each compound was calculated by Origin software. In Table 4, biological reproducibility is reported as the mean IC_50_ SD.

**Evaluation of metabolic rate**

Test compounds (final 50 μM) in DMSO (final 0.5%) were added to a suspension of human hepatocytes (HMCS1S, Thermo Fisher Scientific) at a density of 1.0×10^6^ cells/mL. The cells were incubated at 37 °C in humidified incubator (5% CO_2_) for 2 h, and then acetonitrile was added to stop the reaction. The mixture was vortexed and centrifuged at 15000 rpm for 10 min. HPLC analysis of the supernatant was performed on an analytical column (ODS reversed-phase column, 5 μm, 4.6 mm x 150 mm) eluted with a mobile phase consisting of H_2_O and CH_3_CN at a flow rate of 1.0 mL/min at 37 °C, with UV monitoring at 254 nm. Because some analogs were hydrolyzed in the absence of hepatocytes, we also evaluated the hydrolysis rate without hepatocytes.

**Table S1.** Hydrolysis rate of prodrugs with and without hepatocytes.

| Prodrug | Hydrolysis rate with hepatocytes (%) | Hydrolysis rate without hepatocytes (%) |
| --- | --- | --- |
| **1** | 100 | 14.8 |
| **3a** | 100 | 15.8 |
| **3b** | 92.1 | 6.3 |
| **3c** | 94.5 | 0 |
| **3d** | 77.7 | 0 |
| **4a** | 100 | 0 |
| (*R*)-**4c** | 100 | 13.8 |
